# Comparison of two suspension injection tracers of nano activated carbon and methylene blue in mapping and tracing of sentinel lymph nodes of patients with endometrial cancer

**DOI:** 10.12669/pjms.38.6.5714

**Published:** 2022

**Authors:** Weifeng Zhang, Yan Shu, Dongmei Ni, Zeqiu Wan, Shuying Yang

**Affiliations:** 1 Weifeng Zhang, Department of Gynecology, Huzhou Maternity & Child Health Carel Hospital, Huzhou 313000, Zhejiang Province, P.R. China; 2 Yan Shu, Department of Ultrasound, Huzhou Maternity & Child Health Carel Hospital, Huzhou 313000, Zhejiang Province, P.R. China; 3 Dongmei Ni, Department of Gynecology, Huzhou Maternity & Child Health Carel Hospital, Huzhou 313000, Zhejiang Province, P.R. China; 4 Zeqiu Wan, Department of Gynecology, Huzhou Maternity & Child Health Carel Hospital, Huzhou 313000, Zhejiang Province, P.R. China; 5 Shuying Yang, Department of Gynecology, Huzhou Maternity & Child Health Carel Hospital, Huzhou 313000, Zhejiang Province, P.R. China

**Keywords:** Methylene blue, Nano activated carbon, Endometrial carcinoma, Sentinel lymph nodes, Development, Tracer time

## Abstract

**Objectives:**

To analyze the application efficiency of two tracers for sentinel lymph node mapping in patients with endometrial cancer.

**Methods:**

The records of endometrial cancer patients treated in our hospital from July 2019 to July 2021 were selected. Among them, 29 patients received methylene blue suspension injection and 33 patients received nano activated carbon suspension injection. The staining of sentinel lymph nodes was recorded and the application efficiency of two different tracers were analyzed.

**Results:**

Total detection rate, average number of sentinel lymph nodes and bilateral detection rate of nano activated carbon suspension injection were significantly higher than those of methylene blue suspension injection (P<0.05). Detection accuracy, positive predictive value and sensitivity of nano activated carbon suspension injection were significantly higher than those of methylene blue suspension injection (P<0.05). Incidence of complications was the same in the two groups (P>0.05). Tracing time of nano activated carbon suspension injection was significantly lower than that of methylene blue suspension injection, and the total duration was significantly higher than that of methylene blue suspension injection (P<0.05).

**Conclusion:**

Nano activated carbon can obtain good detection effect in sentinel lymph node recognition in endometrial cancer patients, with shorter tracing time and higher total duration than methylene blue suspension.

## INTRODUCTION

Endometrial cancer belongs to a group of epithelial malignant tumors, and occurs in the endometrium of patients, mostly postmenopausal and perimenopausal women. It occurs with the incidence of about 200 thousand new cases every year and is considered the third gynecological malignant tumor causing death after ovarian and cervical cancers,^1^,^2^ and second among the overall malignant tumors of female reproductive system.^3^ The occurrence of the disease is closely related to the lifestyle of patients and differs in various regions.^4^ Common clinical symptoms include abdominal mass, vaginal drainage, irregular vaginal bleeding and pain.^5^

The main treatment for the endometrial cancer is staged surgery, including abdominal paraaortic lymphadenectomy, pelvic lymphadenectomy, double appendectomy and total hysterectomy. Lymph node involvement is the most important diagnostic factor for the initiation of adjuvant therapy. Most patients with this disease are treated with early hysterectomy, simultaneous removal of bilateral accessories, sometimes combined with abdominal aorta and pelvic lymph node dissection. However, there is still no consensus on the unified treatment mode and scope.^6^ Early sentinel lymph node detection and local selective lymph node resection can significantly improve the success rate of operation and overall prognosis.^7^ There is a variability in the practical efficiency of different tracers for sentinel lymph node mapping.^8^ It important, therefore, to identify tracers with the highest accuracy, that are also fast and safe.^9^ In this study, the sentinel lymph node recognition effects of two tracers were analyzed in patients with endometrial cancer.

## METHODS

Patients with endometrial cancer treated in our hospital from July 2019 to July 2021 were selected as the research object, and the patient’s medical records were analyzed. The ethics committee of our hospital approved the study (No. HZFY-L21041865, Date: 2021-Sep-10^th^). A total of 62 patients were collected in this study.

### Inclusion criteria:

• Meets diagnostic criteria of Stage-I and II endometrial cancer;^10^
• Lymph node dissection near abdominal aorta and pelvic cavity;
• Complete clinical data available.

### Exclusion criteria:

• History of pelvic surgery;
• Serious mental illness;
• Patients with other malignancies.

Patients were divided into two groups (methylene blue suspension injection group and nano activated carbon suspension group) based on the injected tracer. Tracer injection was performed following the same procedure for the patients. Briefly, patients were given general anesthesia, followed by routine disinfection and towel laying, routine exploration of the abdominal cavity and pelvic cavity, exposure of the cervix, and gauze pad application to protect the surrounding tissues and organs. For methylene blue suspension injection, 4ml of methylene blue suspension was selected as the tracer. For nano activated carbon suspension injection, patients were administered 3ml of nano activated carbon suspension. The anterior surface of the middle part of the cervix, the midpoint of the posterior wall and the midpoint of the anterior wall were selected as the injection sites, and each site was injected with 1ml of nano activated carbon suspension. The injection points were compressed and electrocoagulated to prevent dye leakage. After the injection, the patient’s peritoneum was moved backward to expose the drainage areas of pelvic lymph nodes such as common iliac, abdominal aorta, internal iliac and inguinal. Stained lymph vessels were dissected along their direction, and the first stained lymph nodes were explored. The number and location of lymph nodes were recorded in detail. Pathological specimens of other lymph nodes and stained lymph nodes were obtained and sent for examination separately. The condition of uterus, peritoneum, aorta and uterine appendages were monitored, and lymph node dissection was carried out. After the operation, other lymph node specimens and sentinel lymph node specimens were sent to pathological examination.

Evaluation of sentinel lymph nodes tracing was performed as follows: the number of cases detected in both hemi-pelvises and the total number of cases detected were recorded, and the detection rates were compared (detection rate = (number of cases detected/total number of cases) ×%). The following parameters were evaluated: average number of detected cases in patients was recorded; ii) effectiveness of tracing methods (accuracy = (number of true positive cases + number of false positive cases)/total number of cases ×%); iii) predictive value (positive predictive value = number of true positive cases/ (number of true positive cases + number of false negative cases) ×%); iv) sensitivity (sensitivity = number of true positive cases/(number of true positive cases + number of false negative cases) ×%); v) complications (infection, chylous fistula and effusion). Tracing time and total duration of the procedure was recorded.

### Statistical Analysis

SPSS 22.0 software was used for data analysis. The measurement data were expressed in (±*s*) and t-test was performed. The counting data were expressed by n [%], and the test was done. *P*< 0.05 was considered statistically significant.

## RESULTS

A total of 62 patients met the inclusion criteria. Of them, 29 patients, aged 22-58 years, with an average age of (50.31 ± 8.71) years, received methylene blue suspension injection. Thirty-three patients aged 23-59 years, with an average age of (51.03 ± 7.71) years, received nano activated carbon suspension injection. There was no difference in the general characteristics of the two groups (P>0.05). Total detection rate, average number of sentinel lymph nodes and bilateral detection rate of nano activated carbon suspension injection method were significantly higher than those of methylene blue suspension injection (P<0.05) (Table-I).

**Table I:** Comparison of sentinel lymph node identification with two tracers [n(*x̅*±s)].

Tracer	n	Total detection rate (%)	Average number of detected pieces (PCS)	Bilateral detection rate (%)
Nano activated carbon	33	31(93.94)	5.21±1.86	30(90.91)
Methylene blue	29	22(75.86)	4.03±2.44	20(68.96)
*t*	/	4.065	2.148	4.762
*p*	/	0.044	0.036	0.029

The detection accuracy, positive predictive value and sensitivity of nano activated carbon suspension injection were significantly higher than those of methylene blue suspension injection (P<0.05) (Table-II). The incidence of complications of the two tracers were similar, with no statistically significant difference between groups (P>0.05) (Table-III).

**Table II:** Comparison of effectiveness of two tracer tracing methods [n (%)].

Tracer	n	Accuracy Rate	Positive predictive value	Sensitivity
Nano activated carbon	33	100.00	96.97	96.97
Methylene blue	29	86.20	82.76	79.31
*t*	/	4.866	4.806	4.806
*p*	/	0.027	0.028	0.028

**Table III:** Comparison of complications of two tracers [n (%)].

Tracer	n	Infected	Chylous fistula	Effusion	Incidence rate
Nano activated carbon	33	1	1	0	6.1
Methylene blue	29	1	1	1	10.34
	/	/	/	/	0.382
P	/	/	/	/	0.563

Tracing time of nano activated carbon suspension injection was significantly lower than that of methylene blue suspension injection, and the total duration was significantly higher than that of methylene blue suspension injection (P<0.05) (Table-IV).

**Table IV:** Comparison of sentinel lymph node identification with two tracers [n(*x̅*±s)].

Tracer	n	Tracer time(s)	Total duration (h)
Nano activated carbon	33	16.48±2.51	25.51±3.46
Methylene blue	29	37.89±4.00	1.79±0.77
t	/	25.579	3.141
P	/	P<0.001	P<0.001

## DISCUSSION

The results of this study showed that the total detection rate, average number of sentinel lymph nodes and bilateral detection rate, achieved with nano activated carbon suspension injection were significantly higher than those of methylene blue suspension injection (P<0.05). Nano activated carbon suspension injection resulted in significantly higher detection accuracy, positive predictive value and sensitivity as compared to methylene blue suspension injection (P<0.05). The incidence of complications was similar in both groups (P>0.05), while the tracing time of nano activated carbon suspension injection was significantly lower than that of methylene blue suspension injection. At the same time, total duration was significantly higher than that of methylene blue suspension injection (P<0.05). Li Y et al.^11^ conducted a meta-analysis to explore the ability of carbon nanoparticles (CNS) to identify lymph nodes and protect parathyroid gland in thyroid cancer surgery. This meta-analysis identified 11 randomized controlled trials and four non-randomized controlled trials, with a total of 1055 patients. Compared with methylene blue, the use of CNS resulted in an average of 1.50-times more lymph nodes removed per patient (weighted mean difference = 1.50,95% CI = 0.11-2.89, P = 0.03). Accidental parathyroidectomy rate decreased by 5% (odds ratio = 0.05, 95% CI = 0.01-0.29, P = 0.0007). In addition, Liu F et al.^12^ compared the effects of methylene blue and nano carbon in sentinel lymph nodes (SLN) of thyroid papillary carcinoma (PTC) and showed that, although there was no significant difference in the detection number of the two tracers (the detection number of activated carbon nanoparticle suspension was 46 and the detection number of methylene blue was 43), the error rate of parathyroidectomy and the incidence of temporary hypoparathyroidism in methylene blue group were significantly higher than those in nano carbon group (t = 4.137,P<0.05). These results suggest that selecting nano activated carbon as tracer can obtain better sentinel lymph node recognition effect. When studying and mapping the role of anterograde neuroanatomical tracking technology in the complex interrelationship of nervous system, Liu Y et al.^13^ described a new biotinylated dextran amine (BDA) and red fluorescent carbonized polymer dot (CPD) fluorescent nano nerve tracer. Compared with the traditional tracer, BDA-CPD has the advantages of low toxicity, high photoluminescence intensity, good photostability and simplicity of application. The results of our study also show that the tracing time of nano activated carbon suspension injection is shorter and the total duration is significantly prolonged, which allows to shorten the overall surgical treatment time of patients and improve the surgical outcome.

At present, the commonly used sentinel lymph node detection methods in clinic mainly include three types: radionuclide tracing method, dye method and combined test method. Among them, radionuclide tracing method is associated with a high cost, and relevant imaging equipment and detectors need to be used in the test process. The actual operation of combined test method is complex and requires special instruments for auxiliary inspection. Compared with single tracer method, the cost of the combined test method is higher. Therefore, at this stage, fluorescent dye method is considered a key research topic. Compared with the traditional blue dye test, this test method can significantly improve the bilateral detection rate of sentinel lymph nodes. However, this test method also needs special imaging equipment and fluorescent imaging technology.^14^,^15^ Nano carbon has a significant affinity to the lymphatic system. It allows to quickly label and trace the tested lymph nodes without entering the patient’s blood circulation system.^16^ For patients with endometrial cancer, sentinel lymph node identification using methylene blue and nano carbon as tracers is an efficient method with high sensitivity, negative predictive value and accuracy, high safety and low probability of complications.

He J et al. conducted a study to evaluate whether the use of methylene blue or activated carbon nanoparticles as tracers can increase the number of lymph nodes detected in systematic lymph node dissection tissues in video-assisted thoracoscopic surgery (VATS) for non-small cell lung cancer.^17^ Compared with methylene blue, nano activated carbon showed easier clinical operation and higher stability. Nevertheless, operators need to have specific technical expertise to improve the total detection rate of sentinel lymph nodes,^18^ and the appropriate way of analysis and selection of the two tracers needs to be adjusted according to the specific situation. Sentinel lymph nodes of endometrial cancer patients are mainly located in obturator lymph node area and extrailiac lymph node area. This can be used as a reference to improve the detection rate and accuracy of sentinel lymph nodes mapping when carrying out sentinel lymph node test for patients.

### Limitations of the study

Total detection rates of the two tracers for sentinel lymph nodes are similar, but due to the small sample size of this study and other factors, our results must be taken with caution. To further verify the accuracy of the test results, it is necessary to expand the sample size and to conduct further studies on a large sample size.

## CONCLUSION

Nano activated carbon can obtain good detection effect in the mapping and tracing of sentinel lymph nodes in patients with endometrial cancer. It shows shorter tracing time and longer total duration compared to methylene blue tracer. This study provides basis for more effective and comprehensive approach to clinical treatment of patients with endometrial cancer.

### Authors’ contributions:

**WZ:** Conceived and designed the study.

**YS, DN, ZW and SY:** Collected the data and performed the analysis.

**WZ:** Was involved in the writing of the manuscript and is responsible for the integrity of the study.

All authors have read and approved the final manuscript.

## References

[1] Sorosky JI (2012). Endometrial cancer. Obstet Gynecol.

[2] Batool S, Manzur S, Raza S (2013). Accuracy of Doppler ultrasound in diagnosis of endometrial carcinoma. J Pak Med Assoc.

[3] Liu J, Mei J, Li S, Wu Z, Zhang Y (2020). Establishment of a novel cell cycle-related prognostic signature predicting prognosis in patients with endometrial cancer. Cancer Cell Int.

[4] Urick ME, Bell DW (2019). Clinical actionability of molecular targets in endometrial cancer. Nat Rev Cancer.

[5] Doherty MT, Sanni OB, Coleman HG, Cardwell CR, McCluggage WG, Quinn D (2020). Concurrent and future risk of endometrial cancer in women with endometrial hyperplasia:A systematic review and meta-analysis. PLoS One.

[6] Corzo C, Barrientos Santillan N, Westin SN, Ramirez PT (2018). Updates on Conservative Management of Endometrial Cancer. J Minim Invasive Gynecol.

[7] Passarello K, Kurian S, Villanueva V (2019). Endometrial Cancer:An Overview of Pathophysiology, Management, and Care. Semin Oncol Nurs.

[8] Bodurtha Smith AJ, Fader AN, Tanner EJ (2017). Sentinel lymph node assessment in endometrial cancer:A systematic review and Meta-analysis. Am J Obstet Gynecol.

[9] Ahmed M, Purushotham AD, Horgan K, Klaase JM, Douek M (2015). Meta-analysis of superficial versus deep injection of radioactive tracer and blue dye for lymphatic mapping and detection of sentinel lymph nodes in breast cancer. Br J Surg.

[10] Mubarak F, Akhtar MW, Gul-e-Khanda null, Husen YA (2009). Staging of endometrial carcinoma by magnetic resonance imaging:Correlation with surgery and histopathology. J Pak Med Assoc.

[11] Li Y, Jian W-H, Guo Z-M, Li Q-L, Lin S-J, Huang H-Y (2015). A Meta-analysis of Carbon Nanoparticles for Identifying Lymph Nodes and Protecting Parathyroid Glands during Surgery. Otolaryngol Head Neck Surg.

[12] Liu F, Zhu Y, Qian Y, Zhang J, Zhang Y, Zhang Y (2017). Recognition of sentinel lymph nodes in patients with papillary thyroid cancer by nano-carbon and methylene blue. Pak J Med Sci.

[13] Liu Y, Liu J, Zhang J, Li X, Lin F, Zhou N (2019). A brand-new generation of fluorescent nano-neural tracers:biotinylated dextran amine conjugated carbonized polymer dots. Biomater Sci.

[14] Dell'Oglio P, de Vries HM, Mazzone E, Kleinjan GH, Donswijk ML, van der, Poel HG (2020). Hybrid Indocyanine Green-99mTc-nanocolloid for Single-photon Emission Computed Tomography and Combined Radio- and Fluorescence-guided Sentinel Node Biopsy in Penile Cancer:Results of 740 Inguinal Basins Assessed at a Single Institution. Eur Urol.

[15] Lawrie TA, Patel A, Martin-Hirsch PP, Bryant A, Ratnavelu ND, Naik R (2014). Sentinel node assessment for diagnosis of groin lymph node involvement in vulval cancer. Cochrane Database Syst Rev.

[16] Ren W, Chen S, Liao Y, Li S, Ge J, Tao F (2019). Near-infrared fluorescent carbon dots encapsulated liposomes as multifunctional nano-carrier and tracer of the anticancer agent cinobufagin in vivo and in vitro. Colloids Surf B Biointerfaces.

[17] He J, Li S, Shao W, Wang D, Chen M, Yin W (2010). Activated carbon nanoparticles or methylene blue as tracer during video-assisted thoracic surgery for lung cancer can help pathologist find the detected lymph nodes. J Surg Oncol.

[18] Cai H-K, He H-F, Tian W, Zhou M-Q, Hu Y, Deng Y-C (2012). Colorectal cancer lymph node staining by activated carbon nanoparticles suspension in vivo or methylene blue in vitro. World J Gastroenterol.

